# Technology Use and Hospitalization Risk in Older Adults with and without Diabetes

**DOI:** 10.1093/geroni/igaf122.3852

**Published:** 2025-12-31

**Authors:** Mohammed Alqurashi, Martha Coates, Rose Ann DiMaria-Ghalili

**Affiliations:** Drexel University, Alexandria, Virginia, United States; Drexel University, Philadelphia, Pennsylvania, United States; Drexel University, Drexel University, Pennsylvania, United States

## Abstract

Hospitalizations are a significant concern among older adults with diabetes. Technology-related health, including online prescription refills, telehealth visits, and internet health information seeking, are becoming more prevalent, it is important to examine their impact on hospitalization risk. This study investigated the association between digital health technology use and overnight hospitalization in the past year among community-dwelling older adults aged 65 and older, with and without diabetes. We utilized the National Health and Aging Trends Study Round 13 (2023) dataset and the sample included 7,049 community-dwelling older adults aged 65 and older categorized by diabetes status (n = 2,159 with diabetes, n = 4,890 without diabetes). Overnight hospitalization in the last year was reported by 508 (23.6%) of diabetic participants and 801 (16.4%) of non-diabetic participants (p < 0.001). Logistic regression analysis indicated that diabetes was significantly associated with increased odds of hospitalization (OR = 1.57, 95% CI: 1.36–1.81, p < 0.001). Compared to those without diabetes, participants with diabetes were significantly less likely to use computers or tablets (p < 0.001) and telehealth services (p = 0.02), but more likely to refill prescriptions online (p < 0.001). No significant difference was found between groups in using the internet to search for health information (p = 0.34). These findings show that older adults with diabetes are more likely to be hospitalized and less likely to use some digital health tools. Health technology use did not affect hospitalization odds, but further research is needed to explore its impact on outcomes in older adults with diabetes.

